# Climate Change Winners: Receding Ice Fields Facilitate Colony Expansion and Altered Dynamics in an Adélie Penguin Metapopulation

**DOI:** 10.1371/journal.pone.0060568

**Published:** 2013-04-03

**Authors:** Michelle A. LaRue, David G. Ainley, Matt Swanson, Katie M. Dugger, Phil O′B. Lyver, Kerry Barton, Grant Ballard

**Affiliations:** 1 Department of Earth Sciences, Polar Geospatial Center, University of Minnesota, St. Paul, Minnesota, United States of America; 2 HT Harvey and Associates, Los Gatos, California, United States of America; 3 Department of Fisheries and Wildlife, Oregon Cooperative Fish and Wildlife Research Unit, U.S. Geological Survey, Oregon State University, Corvallis, Oregon, United States of America; 4 Landcare Research, Lincoln, New Zealand; 5 Bartonk Solutions, Nelson, New Zealand; 6 PRBO Conservation Science, Petaluma, California, United States of America; Institute of Ecology, Germany

## Abstract

There will be winners and losers as climate change alters the habitats of polar organisms. For an Adélie penguin (*Pygoscelis adeliae*) colony on Beaufort Island (Beaufort), part of a cluster of colonies in the southern Ross Sea, we report a recent population increase in response to increased nesting habitat as glaciers have receded. Emigration rates of birds banded as chicks on Beaufort to colonies on nearby Ross Island decreased after 2005 as available habitat on Beaufort increased, leading to altered dynamics of the metapopulation. Using aerial photography beginning in 1958 and modern satellite imagery, we measured change in area of available nesting habitat and population size of the Beaufort colony. Population size varied with available habitat, and both increased rapidly since the 1990s. In accord with glacial retreat, summer temperatures at nearby McMurdo Station increased by ∼0.50°C per decade since the mid-1980s. Although the Ross Sea is likely to be the last ocean with an intact ecosystem, the recent retreat of ice fields at Beaufort that resulted in increased breeding habitat exemplifies a process that has been underway in the Ross Sea during the entire Holocene. Furthermore, our results are in line with predictions that major ice shelves and glaciers will retreat rapidly elsewhere in the Antarctic, potentially leading to increased breeding habitat for Adélie penguins. Results further indicated that satellite imagery may be used to estimate large changes in Adélie penguin populations, facilitating our understanding of metapopulation dynamics and environmental factors that influence regional populations.

## Introduction

The adage is that global climate change will identify both winners and losers as the habitats of polar organisms are altered [1]. For instance, on one hand, as sea ice extent decreases some species may benefit from increased open-water conditions (i.e., salps [*Salpa thompsoni*], gentoo penguins [*Pygocelis papua*], cryptophytes) [2], [3]; on the other hand, other species may be negatively impacted by a loss of breeding habitat, such as emperor penguins (*Aptenodytes fosteri*) [4], [5], [6]. Indeed, in areas of the Antarctic where sea ice is declining (i.e., the Peninsula), the food web has been in flux, as noted by recent studies [2], [3]. However, on the opposite side of the continent, the Ross Sea (located approximately 3,500 km south of New Zealand), is a unique body of water that has been relatively untouched by human activities, and is likely to provide the last sea-ice ecosystem during the present period of climate change [7]. Until recently, the food web has been little exploited; there are no invasive species, no widespread chemical pollution, and no mineral extraction activities [8], [9], [10]. Therefore, the climate patterns exhibited are likely without complications brought on by many other direct, anthropogenic factors. In the Ross Sea region, changing weather patterns have brought slightly warmer temperatures and stronger winds, with corresponding increases in sea ice extent and persistence [11], [12], [13] and more predictable coastal polynyas [9], [11], [14].

**Figure 1:**
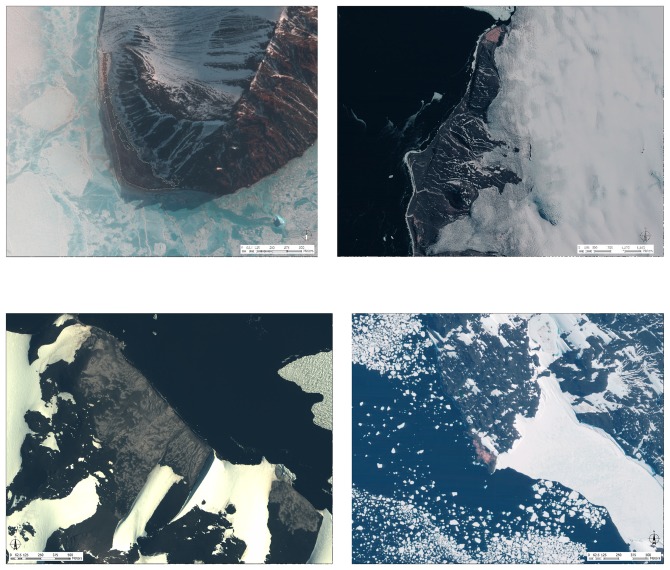
Satellite images of each Adélie penguin colony within the 4-colony metapopulation in the southern Ross Sea showing colony area at each location. Clockwise from top left: Beaufort, which has been habitat-limited by steep moraines to the east, a glacier to the north, and the ocean to the west and south; Bird, with a glacier to the east; Royds, with fast ice to the southeast; and Crozier colonies, both east and west, that are separated by a glacial field. Images are QuickBird-2, courtesy DigitalGlobe, Inc.

As a result of the earlier-opening and longer-lasting polynyas, the Adélie penguin (*Pygoscelis adeliae*) colonies along the Ross Sea coast grew during the 1980s–90s, affecting almost 40% of the world population (approximately 2.5 million breeding pairs) [9], [15], [16]. While that rapid population growth has ceased, we report here more recent changes in the Beaufort Island (herein referred to as Beaufort) colony, part of a four-colony cluster that includes 10% of the world population of Adélie penguins [16]. The remaining colonies in this cluster, at Capes Royds, Bird, and Crozier (herein referred to as Royds, Bird and Crozier), are located on nearby Ross Island [17]. These colonies and others within the Ross Sea are sensitive to ice sheet and glacier retreat, according to analysis of subfossil remains deposited through the Holocene and back to the previous interglacial period [18], [19]. The colonies on Ross and Beaufort islands are the youngest colonies in the Ross Sea [18], and among these Beaufort has been the only habitat-limited colony in the metapopulation, as it sits upon a gravel moraine hemmed in by cliffs and glaciers (Figure 1).

Therefore, we hypothesized that a recently observed increase in breeding pairs and availability of nesting habitat at Beaufort was associated with glacial retreat, and explained a concurrent reduction in emigration from Beaufort to nearby colonies on Ross Island. Our study objectives were to: 1) estimate available habitat and population size of an Adélie penguin colony at Beaufort; 2) calculate change in available habitat and glacial retreat during 1958–2010 (the period for which images are available); and 3) better understand the dynamics within the Ross-Beaufort islands metapopulation.

## Methods

**Figure 2:**
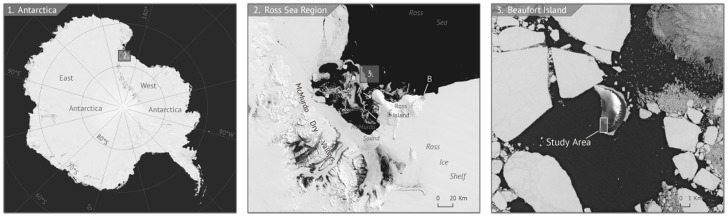
Locator map for our study area at Beaufort Island, Ross Sea, Antarctica. Left, the location of the Ross Sea region; middle, the location of Beaufort Island and the locations of other Adélie penguin colonies on nearby Ross Island (A. Cape Bird, B. Cape Crozier, C. Cape Royds) and, right, the location of the main Adélie penguin colony on Beaufort Island.

The main Adélie penguin colony at Beaufort is located at the island's southwest coast (∼76° 58′ S and ∼166° 54′ E), approximately 20 km and 50 km north of Bird and Royds, respectively, and 40 km west of Crozier, all on Ross Island (Figure 2). To estimate population size (i.e., breeding pairs) at Beaufort during 1983–2010, we counted individual nesting territories using aerial photographs taken approximately 800 m above ground level just after onset of incubation (courtesy Landcare Research, New Zealand). We define “nesting territories” as sites occupied and defended by adults during the egg-laying and early incubation periods. We used photographs that were taken each year as close as possible to 1 December, a date on which the colony population was represented almost entirely by one member of each penguin pair incubating its eggs, and minimal numbers of non-breeders not on territories [15].

**Figure 3:**
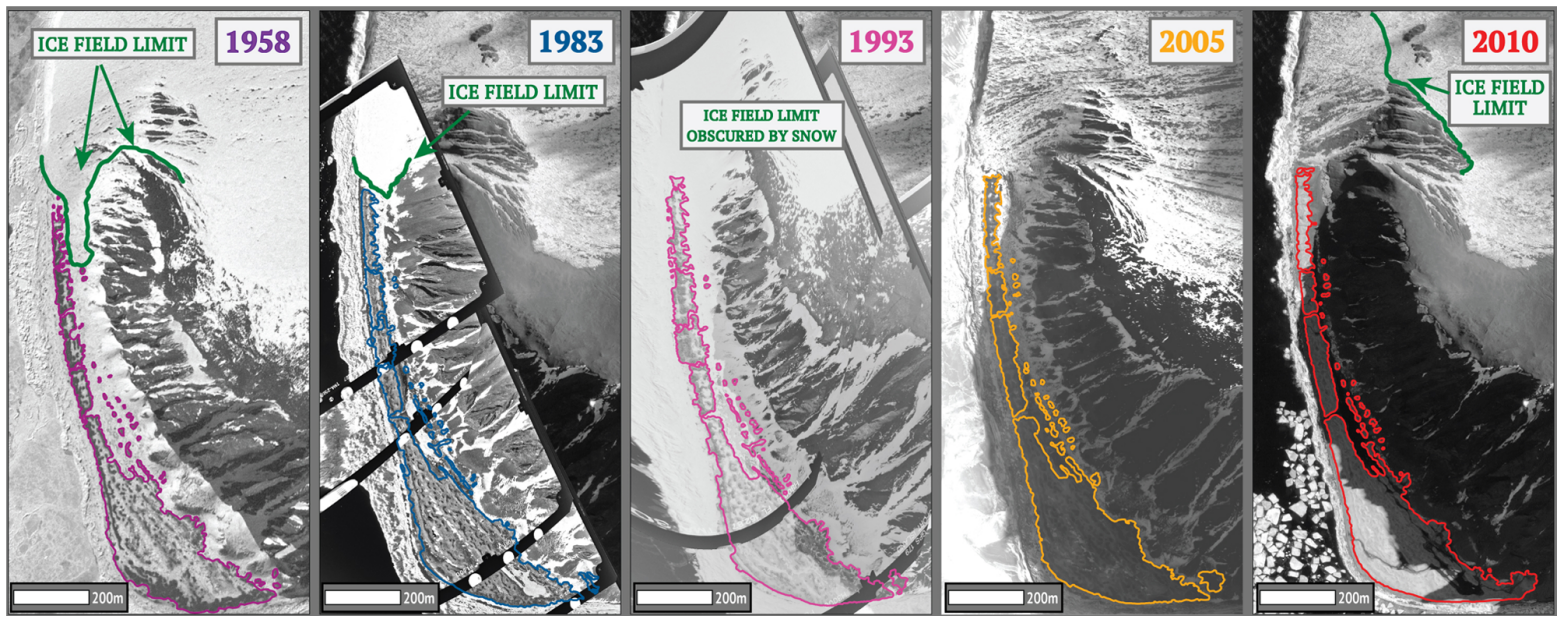
Changes in snow and ice cover and spatial extent of the Adélie penguin colony at the southern end of Beaufort Island, Antarctica, from 1958–2010 using air photos and high-resolution (0.6 m) satellite imagery (copyright DigitalGlobe, Inc). During the early years, best seen in the 1958 and 1993 image, snow covered the area, with penguins nesting on bare mounds and ridges.

To determine changes in available nesting habitat, we gathered aerial photos taken during the penguin incubation period in 1958, 1983, and 1993 (0.19–0.38 m resolution; USGS, US Navy), and high-resolution satellite images from 2005 and 2010 (0.6 m resolution; copyright DigitalGlobe, Inc.). In ArcGIS 10, we georeferenced images with tie points (e.g., boulders, cliff peaks) on Beaufort to overlay images exactly. Differences between image resolutions meant we were unable to directly delineate actual nest space. Instead, we calculated available habitat (m^2^) for the colony per image year (Figure 3) by tracing the maximum extent of the current-year guano stain and subtracting area of unsuitable habitat (i.e., snow and ice cover) within colony boundaries. We interpreted the current-year guano stain by viewing panchromatic (i.e., grayscale) images; active guano areas had a brighter spectral signature than rock or remnant guano stains. We also delineated the edge of the ice field to the north of the colony (Figure 3) on each image to understand decadal environmental changes.

We addressed changes in movement of penguins between Beaufort and colonies on Ross Island by marking 400 near-to-fledging chicks per year at Beaufort during 1999–2010 (excluding 2005 and 2008, when we could not reach Beaufort at the appropriate time of the season). We then intently searched for banded birds at Ross Island colonies in subsequent years (to 2011) and recorded band numbers and reproductive status [20]. Each colony was completely searched every 2–7 days throughout the breeding season, and high annual resighting probabilities of banded birds (>70%) indicate the comprehensive nature of the effort [20]. Problems of access to Beaufort meant we could not band-search there sufficiently enough each year to estimate survival or detection rates via mark-recapture directly [20], [21]. However, we used age-related survival rates from Bird and Crozier to adjust the number of Beaufort-banded individuals within each age-cohort each year that should have been alive and detectable at Royds, Bird, or Crozier during 2001–2011. The result is the proportion of banded Beaufort birds seen at one of the other three colonies, relative to the total number of Beaufort-banded birds potentially alive each year; here we define this as a measure of the “emigration rate” from Beaufort to other colonies.

Finally, to address changing weather patterns, we gathered all available temperature records from McMurdo Station (available at http://www.antarctica.ac.uk/met/READER/), which is located on Ross Island. We then calculated changes in annual summer (averaged monthly November-February) temperatures during 1958–2010.

## Results

**Figure 4:**
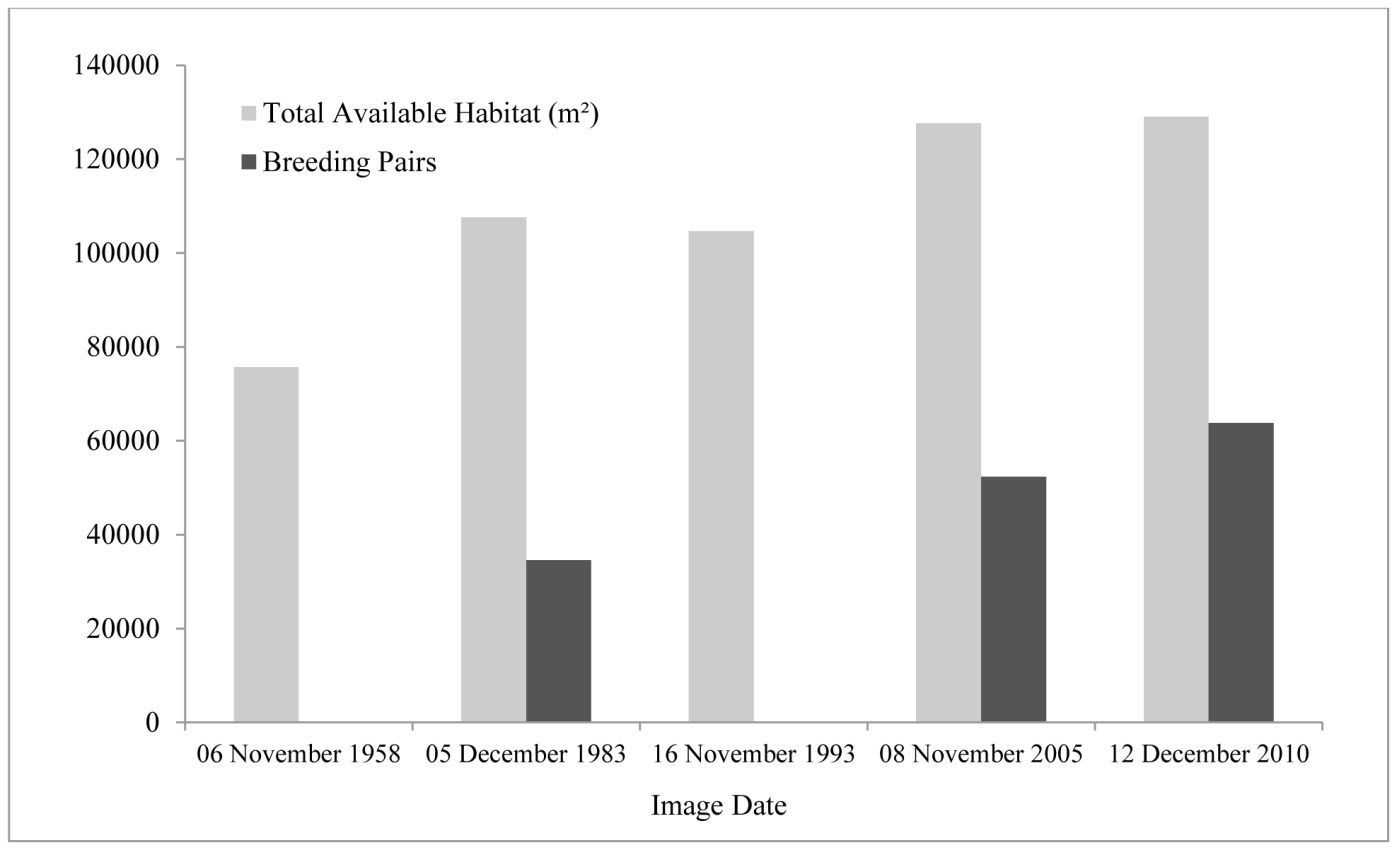
Available habitat (m^2^) and breeding pairs of Adélie penguins at the main Beaufort Island colony during 1958–2010. Available habitat was defined as the maximum extent of the guano stain of the colony minus the snow cover (i.e., unsuitable habitat) within the colony boundary.

Available habitat for Adélie penguins at the main portion of the Beaufort colony, on the south coast, increased 71% since 1958, with a 20% increase during 1983–2010 (Figure 4). During the same time, population size increased (+84%), as did colony density (0.31–0.49 breeding pairs/m^2^; Table 1).We also found a positive association between colony area and population estimates for years with overlapping data (*n* = 3; Figure 4). The extent of the snow- and ice-field to the north of the main colony did not change from 1958–1983, but then retreated 543 m during 1983–2010 (Figure 3). Further, in 2004 we observed a newly-founded, disjunct subcolony at the northeast coast of Beaufort. Population estimates from aerial photography there indicated a population increase from 460 pairs to 957 pairs by 2010 (change of 108%), and we also found several Beaufort-banded penguins there.

**Table 1:** Total available habitat (m^2^), number of breeding pairs (BP) counted from independent air photos, and calculated density (breeding pairs/m^2^) of the Adélie penguin colony at Beaufort Island, Antarctica, during November/December each year.

Year	Total Available Habitat (m^2^)	Breeding Pairs	Density (BP/m^2^)
1958	75670.3	--	--
1983	107571.2	34588	0.32
1993	104637.3	--	--
2005	127603.4	52335	0.41
2010	129029.5	63760	0.49

**Figure 5:**
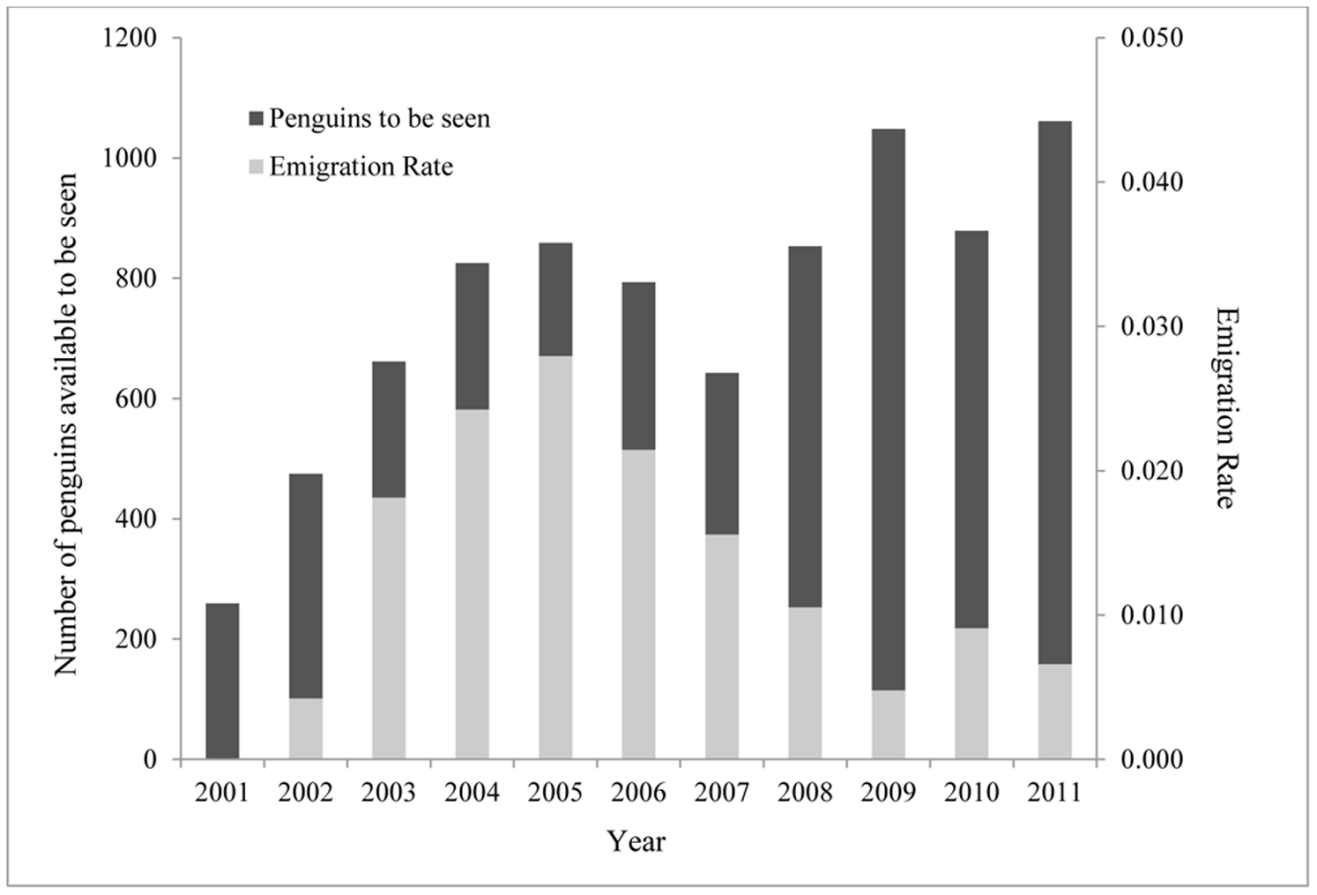
The proportion of banded Beaufort birds seen at one of the other three Ross Island colonies (Capes Royds, Bird, Crozier), relative to the total number of Beaufort-banded birds potentially alive each year (“Emigration Rate”; light bars) and the total number of banded Beaufort birds potentially available (dark bars) during band searches. Except for 2005 and 2008, 400 chicks were banded at Beaufort Island per year from 1999–2010. Birds banded as chicks at the beginning of the study (1999) began returning to breeding colonies within the Ross-Beaufort island metapopluation in 2002.

**Figure 6:**
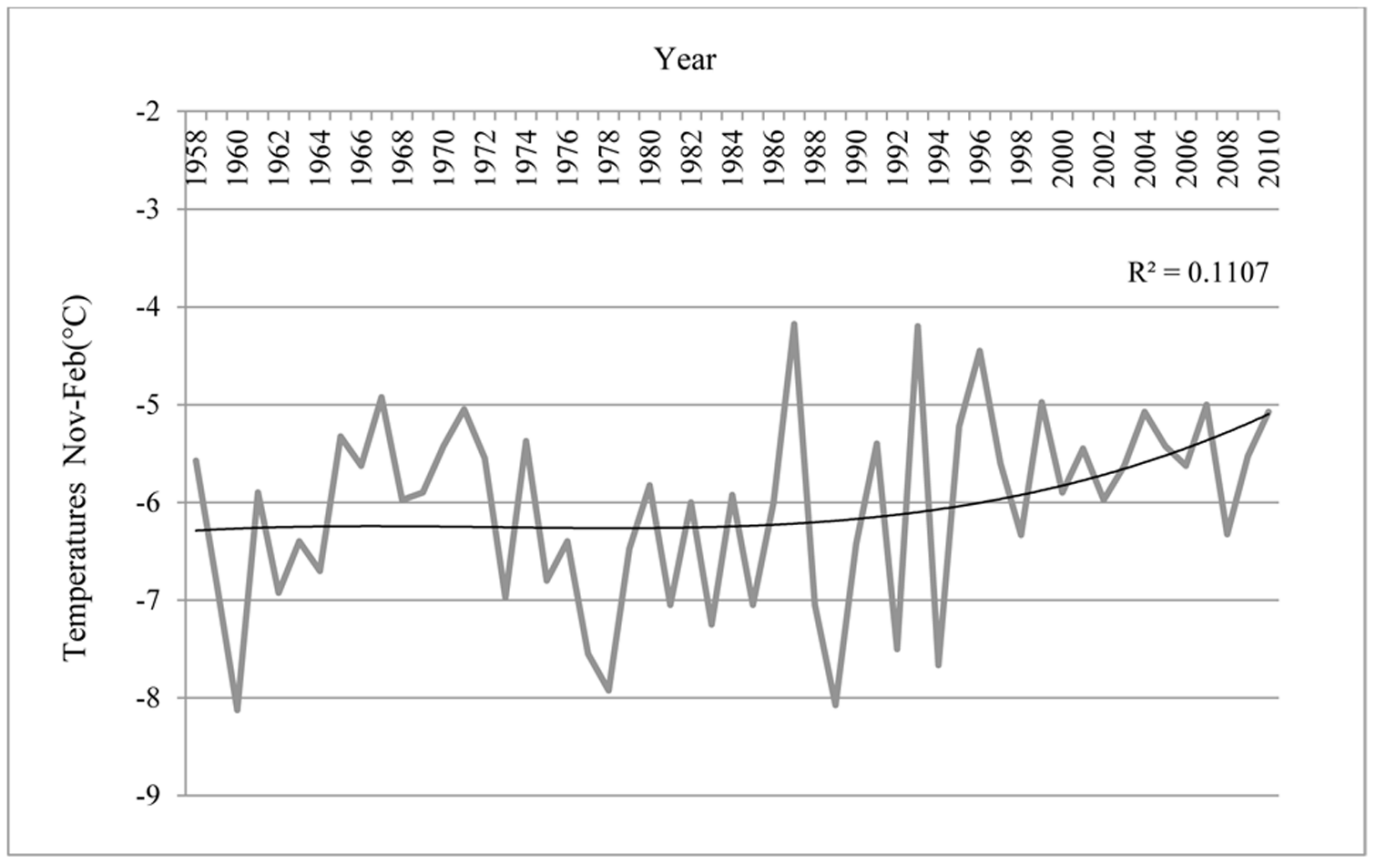
Average summer (November–February) temperatures in °C recorded at McMurdo Station, approximately 90 km south of Beaufort Island, Antarctica, during 1958–2010.

The emigration rate of Beaufort chicks visiting colonies on Ross Island during 2001–2011 (when we were band-searching) increased sharply from nearly zero in 2002 and peaked at 3% in 2005 (Figure 5), despite a relatively stable period of colony size. Subsequently, although more Beaufort birds were available to visit away from Beaufort, visitation of Ross Island colonies decreased markedly after 2005. Finally, as an indication of changing weather patterns, we found that average summer air temperatures recorded at McMurdo Station increased by 1°C during 1958–2010, with most of the increase occurring during 1980–2000 (Figure 6). Average temperatures during October-December, the period of snow melt/ablation within the colony, increased by 3.22°C.

## Discussion

Evidence suggesting that Adélie penguins on Beaufort were “climate change winners” was both the colony expansion, and increases in nesting density and summer temperatures during the 52-year study period. Our result of population expansion is markedly different than what might be expected elsewhere within the Adélie penguin's continental range, where populations are declining due to warming temperatures; for example, on the Antarctic Peninsula [4]. Not only did the glacier field to the north of the main colony retreat by hundreds of meters allowing for colony expansion, but the snow patches (i.e., unsuitable habitat) within the colony decreased and eventually vanished. Both of these small-scale (snow patches) and large-scale (glacial retreat) factors driven, at least in part, by increasing temperatures played a role in the increase of the Adélie penguin nesting habitat and colony size. Indeed, the Adélie penguin population in the greater Ross Sea region has expanded over the last 12,000 years, as glaciers have retreated from positions occupied during the Last Glacial Maximum [18], [19]. The population processes observed at Beaufort and within the Ross-Beaufort metapopulation could be prevalent elsewhere; for instance, perhaps in the southern Antarctic Peninsula where glacial retreat and ice shelf disappearance has recently been particularly rapid [22]. However, especially warm temperatures in that sector have also led to increased snowfall [23] and decreased sea ice, with detrimental impacts on Adélie penguin colonies there [2], [24], [25].

Our results show a response to altered availability of nesting habitat that consequently changed dynamics for the Ross-Beaufort metapopulation, particularly emigration and immigration [26]. The emigration rate of Adélie penguins from Beaufort to nearby colonies was comparable to the highest movement probabilities observed at colonies on Ross Island [20]. However, after 2005 the emigration rates from Beaufort decreased rapidly when glacial retreat accelerated, the main colony increased, and the new subcolony was founded on the north shore. These concurrent results suggest that the pressure to emigrate from the main colony at Beaufort decreased as nesting habitat expanded. Notably, some of the space created by the habitat expansion was unsuitable for nesting (steep terrain) and the glacial retreat seems to have been more rapid than the penguins were capable of accommodating. In other words, our analysis detected an initial population expansion, with additional growth perhaps still underway, providing that the factors leading to the overall population increase are still in place. Importantly, the four-colony, southern Ross Sea metapopulation has been growing again after a stable period in the 1990s [9], but Beaufort only recently has been able to contribute to the population increase as the glacier has retreated. None of the Ross Island colonies are even close to being space limited.

We can only speculate on other environmental and biotic factors that may have played a role in the recent population increase at Beaufort and within the metapopulation. For instance, Adélie penguins of the southern Ross Sea are important predators of crystal krill (*Euphausia crystallorophias*) and silverfish (*Pleuragramma antarctica*) [16], [27], and are also prey of leopard seals (*Hydrurga leptonyx*), with consumption and predation rates varying with colony size [28], [29]. The availability of crystal krill and silverfish could change as the sea ice season and ice cover changes locally (with earlier polynyas), versus regionally (with greater extent and longer seasons) in the Ross Sea sector [12], [13], [30]. However, the direction of that change as possibly driven by trophic factors is currently unknown, because relatively little work has been conducted on those two sea-ice obligate, high-latitude prey species. On the other hand, an industrial fishery recently arrived in the Ross Sea targets a major trophic competitor of Adélie penguins, the Antarctic toothfish (*Dissostichus mawsoni*), which has since declined in prevalence in the region [10]. Both toothfish and penguins prey heavily on silverfish in the southern Ross Sea [27], [30], and it is possible the population increase we report here was partly due to increased silverfish availability. At this stage, we can say little more on the extent to which changed tropho-dynamics are playing a role in the population growth of either Beaufort, or the metapopulation. Nonetheless, glacial retreat and snow melt, increases in available habitat, and subsequent decreases in emigration rates from Beaufort Island indicate that warming temperatures (related to climate change) contributed to a change in metapopulation dynamics of Adélie penguins in the southern Ross Sea region.

Finally, despite only three years for comparison, we were encouraged by the potential association between available habitat and population size at Beaufort. On the basis of our results, we suggest that high-resolution satellite imagery is able to index population size of Adélie penguins at Beaufort, and presumably elsewhere. Indeed, satellite imagery has proven a useful tool for detection and abundance estimation of other polar species [31], [32], [33], [34]. The idea of expanding this technique and remotely assessing Adélie penguin populations is important, given a full census of the global population has never been conducted concurrently, and because this species appears to be especially sensitive to environmental change, which is progressing differently depending on region [16], [4], [35]. Rapid physical changes in the Southern Ocean ecosystem are occurring (e.g., ocean temperatures and salinity, sea ice extent, fishing) [10], [14], [25], [36] and monitoring Adélie penguin numbers, as an indicator species, would be beneficial to gauge how the sea-ice obligate biota are responding. Despite recent evidence indicating that satellite imagery is remarkably accurate in assessing population changes of another population of pygoscelid penguins [34], more research is needed to investigate the lower threshold of variance/validity for indexing Adélie penguin populations with a similar technique. Perhaps only large populations or large changes, as at Beaufort, would be detectable.

## References

[1] Glantz MH (1995) Assessing the impacts of climate: The issue of winners and losers in a global climate change context. Stud Env Sci 65: 41–54.

[2] Ducklow HW, Baker K, Martinson DG, Quetin LB, Ross RM, et al (2007) Marine pelagic ecosystems: the West Antarctic Peninsula. Phil Trans Royal Soc London B 362: 67–94. 10.1098/rstb.2006.1955 PMC1764834 17405208

[3] Montes-Hugo M, Doney SC, Ducklow HW, Fraser WR, Martinson D, et al (2009) Recent changes in phytoplankton communities associated with rapid regional climate change. Science 323: 1470–1473. 19286554 10.1126/science.1164533

[4] Ainley DG, Russel J, Jenouvrier S, Woehler E, Lyver POB, et al (2010) Antarctic penguin response to habitat change as earth's troposphere reaches 2° C above preindustrial levels. Ecol Monogr 80: 49–66.

[5] Jenouvrier S, Caswell H, Barbraud C, Holland M, Stroeves J, et al (2009) Demographic models and IPCC climate projections predict the decline of an emperor penguin population. Proc Natl Acad Sci USA 106: 1844–1847. 19171908 10.1073/pnas.0806638106 PMC2644125

[6] Jenouvrier S, Holland M, Stroeves J, Barbraud C, Weimerskirch H, et al (2012) Effects of climate change on an emperor penguin population: analysis of coupled demographic and climate models. Glob Change Biol 18: 2756–2770. 10.1111/j.1365-2486.2012.02744.x 24501054

[7] Stammerjohn SE, Massom R, Rind D, Martinson DG (2012) Regions of rapid sea ice change: An inter-hemispheric seasonal comparison. Geo Res Let 39: L06501 doi:10.1029/2012GL050874.

[8] Ainley DG (2002) The Ross Sea, Antarctica, where all ecosystem processes still remain for study, but maybe not for long. Mar Ornithol 30: 55–62.

[9] Ainley DG, Ballard G, Weller J (2010) Ross sea biodiversity part 1: Validation of the 2007 CCAMLR bioregionalization workshop results towards including the Ross Sea representative network of marine protected areas in the Southern Ocean. WG-EMM 10/11: 1–62.

[10] Ainley DG, Nur N, Eastman JT, Ballard G, Parkinson CL, et al (2012) Decadal trends in abundance, size and condition of Antarctic toothfish in McMurdo Sound, Antarctica, 1972–2011. Fish and Fisheries doi:10.1111/j.1467-2979.2012.00474.x.

[11] Ainley DG, Clarke ED, Arrigo K, Fraser WR, Kato A, et al (2005) Decadal-scale changes in the climate and biota of the pacific sector of the Southern Ocean, 1950s to the 1990s. Antarct Sci 17: 171–182.

[12] Parkinson CL (2002) Trends in the length of the Southern Ocean sea-ice season, 1979–99. An Glac 34: 435–440.

[13] Stammerjohn S, Martinson D, Smith R, Yuan X, Rind D (2008) Trends in Antarctic annual sea ice retreat and advance and their relation to el Niño–Southern oscillation and southern annular mode variability. J Geo Res 113. doi: 10.1029/2007JC004269.

[14] Massom RA, Stammerjohn SE (2010) Antarctic sea ice change and variability: Physical and ecological implications. Polar Sci 4: 149–186.

[15] Taylor R, Wilson P (1990) Recent increase and southern expansion of Adélie penguin populations in the Ross Sea, Antarctica, related to climatic warming. N Z J Ecol 14: 25–29.

[16] Ainley DG (2002) The Adélie penguin: bellwether of climate change. New York: Columbia University Press. 310 p.

[17] Ainley DG, Nur N, Woehler EJ (1995) Factors affecting the distribution and size of Pygoscelid penguin colonies in the Antarctic. Auk 112: 171–182.

[18] Emslie SD, Coats L, Licht K (2007) A 45,000 yr record of Adélie penguins and climate change in the Ross Sea, Antarctica. Geol 35: 61–64.

[19] Millar CD, Subramanian S, Heupink TH, Swaninathan S, Baront C, et al (2012) Adélie penguins and temperature changes in Antarctica: a long-term view. Integr Zool 7: 113–120. 22691195 10.1111/j.1749-4877.2012.00288.x

[20] Dugger KM, Ainley DG, Lyver POB, Barton K, Ballard G (2010) Survival differences and the effect of environmental instability on breeding dispersal in an Adélie penguin metapopulation. Proc Natl Acad Sci USA 107: 12375–12380. 20566874 10.1073/pnas.1000623107 PMC2901434

[21] Dugger KM, Ballard G, Ainley DG, Barton KJ, Burger A (2006) Effects of flipper bands on foraging behavior and survival of Adélie penguins (*Pygoscelis adeliae*). Auk 123: 858–869.

[22] Cook AJ, Fox AJ, Vaughan DG, Ferrigo JG (2005) Retreating glacier fronts on the Antarctic Peninsula over the past half-century. Science 308: 541–544. 15845851 10.1126/science.1104235

[23] Turner J, Overland JE, Walsh JE (2007) An Arctic and Antarctic perspective on recent climate change. Int J Clim 27: 277–293.

[24] Fraser WR, Patterson DL (1997) Human disturbance and long-term changes in Adélie penguin populations: a natural experiment at Palmer Station, Antarctica. In Battaglia B, Valencia J, Walton D, editors. Antarctic communities: species, structure and survival. Cambridge: Cambridge University Press. pp. 445–452.

[25] Trivelpiece WZ, Hinke JT, Miller AK, Reiss CS, Trivelpiece SG, et al (2011) Variability in krill biomass links harvesting and climate warming to penguin population changes in Antarctica. Proc Natl Acad Sci USA 108: 7625–7628. 21482793 10.1073/pnas.1016560108 PMC3088573

[26] Shepherd LD, Millar DG, Ballard G, Ainley DG, Wilson PR, et al (2005) Microevolution and mega-icebergs in the Antarctic. Proc Natl Acad Sci USA 102: 16717–16722. 16275908 10.1073/pnas.0502281102 PMC1283793

[27] Ainley DG, Ballard G, Barton KJ, Karl BJ, Rau GH, et al (2003) Spatial and temporal variation of diet within a presumed metapopulation of Adélie penguins. Condor 105: 95–106.

[28] Ainley DG, Ballard G, Karl BJ, Dugger KM (2005) Leopard seal predation rates at penguin colonies of different size. Antarct Sci 17: 335–340.

[29] Ballance LT, Ainley DG, Ballard G, Barton KJ (2009) An energetic correlate between colony size and foraging effort in seabirds, an example of the Adélie penguin *Pygoscelis adeliae* . J Avian Biol 40: 279–288.

[30] La Mesa M, Eastman JT, Vacchi M (2004) The role of notothenioid fish in the food web of the Ross Sea shelf waters: a review. Polar Biol 27: 321–338.

[31] LaRue MA, Rotella JJ, Garrott RA, Siniff DB, Ainley DG, et al (2011) Satellite imagery can be used to detect variation in abundance of Weddell seals (*Leptonychotes weddellii*) in Erebus Bay, Antarctica. Polar Biol 34: 1727–1737.

[32] Fretwell PT, LaRue MA, Morin P, Kooyman GL, Wienecke B, et al (2012) The first global, synoptic survey of a species from space: emperor penguins. PLoS ONE 7(4): e33751 doi:10.1371/journal.pone.0033751. 22514609 10.1371/journal.pone.0033751 PMC3325796

[33] Lynch HJ, White R, Black AD, Naveen R (2012) Detection, differentiation, and abundance estimation of penguin species by high-resolution satellite imagery. Polar Biol 35: 963–968.

[34] Naveen R, Lynch HJ, Forrest S, Mueller T, Polito M (2012) First direct, site-wide penguin survey at Deception island, Antarctica, suggests significant declines in breeding chinstrap penguins. Polar Biol 35: 1879–1888.

[35] Croxall JP, Trathan PN, Murphy EJ (2002) Environmental change and Antarctic seabird populations. Science 297: 1510–1514. 12202819 10.1126/science.1071987

[36] Jacobs S (2006) Observations of change in the Southern Ocean. Phil Trans Royal Soc Ser 364: 1657–1681. 10.1098/rsta.2006.1794 16782605

